# Diagnostic testing and laboratory equity in clinical microbiology

**DOI:** 10.1128/spectrum.01222-24

**Published:** 2024-06-25

**Authors:** Paige M. K. Larkin, Elitza S. Theel, Karissa Culbreath, Omai B. Garner, Shannon Vassell, Wes Kim, Rosemary C. She, S. Wesley Long, N. Esther Babady

**Affiliations:** 1 American Society for Microbiology, Washington, DC, USA; 2 Mayo Clinic Minnesota, Rochester, Minnesota, USA; 3 TriCore, Albuquerque, New Mexico, USA; 4 University of California Los Angeles, Los Angeles, California, USA; 5 City of Hope, Duarte, California, USA; 6 Houston Methodist Hospital, Houston, Texas, USA; 7 Memorial Sloane Kettering Cancer Center, New York, New York, USA; Broad Institute, Cambridge, Massachusetts, USA

## EDITORIAL

Significant advances in diagnostic testing, including next generation sequencing and point-of-care testing (POCT), have led to improved pathogen detection, identification and ultimately patient care. However, neither these emerging technologies nor conventional methods are universally available to all patients due to inequities in the healthcare system. Health equity is the state, in which everyone has a fair and just opportunity to attain their highest level of health, which requires efforts to address health injustice, obstacles to attaining healthcare and prevention of health disparities (1). Health equity is commonly associated with access to preventative care and effective treatment. Another key health equity aspect is laboratory equity, which includes providing accurate, accessible, affordable, and timely diagnostics to inform clinical decisions and improve patient outcomes.

High quality and timely tests may not be broadly accessible due to geographic location (e.g., rural or without access to reliable transportation), cost (e.g., lack of insurance), bias, stigma, or discrimination within a healthcare setting, and/or lack of permanent housing or incarceration, preventing effective follow-up. This is exacerbated for some diseases, and in particular for those that primarily affect vulnerable or disadvantaged populations. For example, testing is either non-existent or significantly limited for pathogens more commonly afflicting unhoused individuals (e.g., *Rickettsia prowazekii, Bartonella quintana*).

The ASM Laboratory Equity Working Group was founded in 2023 with the goal of facilitating sustained advancement of clinical microbiology laboratory practices, within and beyond the US, promoting equitable access to timely and quality-assured infectious disease diagnostic testing. This Working Group will identify challenges that hinder achievements of equity in clinical microbiology laboratories, recognize risks associated with not addressing these challenges and provide recommendations to enhance laboratory equity. The Working Group will facilitate the dissemination of data through peer-reviewed publications in journals, identify and provide recommendations through guidelines to address barriers of lab equity, identify strategies for engaging stakeholders, and establish a network to support individuals dedicated to advancing this essential piece of healthcare delivery.

The ASM Lab Equity Working Group is now partnering with the Editorial leadership at *Microbiology Spectrum* to draw attention to this effort and to increase the visibility of studies that are working toward lab equity. *Microbiology Spectrum* will continue to welcome studies that are focused on improving lab equity across the world. Publication and dissemination of these studies are critical for advancing global equity efforts, and for guiding complementary efforts within specific populations. These manuscripts can serve as guides for implementing new lab practices and allow authors to find each other and collaborate further. Scientists often find it difficult to share these results as not many journals offer formats that accommodate such reports. ASM’s newest journal, *Microbiology Spectrum* already serves as a platform for the publication of such reports, from studies that highlight the difficulty of implementing diagnostic assays in low-resource settings (2–4) to studies focused on prevalence of pathogens in regions lacking robust health and/or diagnostic infrastructure (5, 6). With the publication of this Editorial alongside this Call for Papers, we encourage colleagues across the globe to consider *Microbiology Spectrum* as a venue for the publication of studies that are centered on Lab Equity.
